# Bone turnover marker rhythmicity: implications for osteoporosis treatment and fracture healing

**DOI:** 10.1530/EC-26-0114

**Published:** 2026-06-26

**Authors:** Megan M Tidd, Christine M Swanson

**Affiliations:** ^1^ Department of Orthopedics, University of Colorado Anschutz, Aurora, Colorado, USA; ^2^ Division of Endocrinology, Metabolism and Diabetes, University of Colorado Anschutz, Aurora, Colorado, USA; ^3^ Geriatric Research, Education and Clinical Center, Rocky Mountain Regional VA, Aurora, Colorado, USA

**Keywords:** circadian rhythm, bone turnover markers, chronotherapy, osteoporosis, fracture healing

## Abstract

**Abstract:**

Bone turnover is a dynamic process with markers of bone turnover displaying varying degrees of rhythmicity. The objective of this narrative review was to synthesize current evidence on the regulation of bone turnover marker (BTM) rhythmicity and to evaluate how these rhythms may influence clinically relevant outcomes in osteoporosis treatment and fracture healing. Structured literature searches were conducted in PubMed to identify studies that examined BTM rhythmicity, intrinsic versus extrinsic factors that influence this rhythmicity, and timing effects of osteoporosis medications and fracture-related healing to inform this narrative review. The reviewed evidence demonstrates that markers of bone resorption exhibit robust diurnal rhythms, whereas bone formation markers show lower-amplitude and more variable oscillations. These rhythms are primarily circadian driven and influenced by environmental factors (e.g. fasting) and have important implications for accurate use and interpretation when used clinically and in research. Clinical and preclinical studies further indicate that the administration time of some osteoporosis medications can influence efficacy and safety, with certain anabolic and antiresorptive agents showing differences in biochemical or structural responses depending on administration time, while others appear timing insensitive or remain unstudied. Emerging experimental data suggest that fracture healing may also display rhythmicity and be differentially impacted by the timing of interventions.

**Plain language summary:**

This review summarizes how bone turnover marker levels vary across the day and night and how this rhythmicity may influence the effects of some medications, with implications for optimal use of these markers in clinical practice and research to improve osteoporosis treatment and potentially fracture healing.

## Introduction

Bone turnover is a highly dynamic and tightly regulated process governed by coordinated cycles of bone formation and resorption (1, 2). An expanding body of evidence has demonstrated that this remodeling process is strongly influenced by circadian biology (1, 2, 3, 4, 5, 6, 7). Numerous biochemical bone turnover markers (BTMs) exhibit robust daily sinusoidal patterns that arise from intrinsic circadian clocks but are significantly modified by behavioral and environmental factors, such as feeding, sleep, and activity (3, 4, 6, 7, 8, 9, 10, 11). These oscillations have direct implications for clinical practice, affecting the acquisition and interpretation of laboratory values, the design of studies, and the timing of therapeutic interventions.

Parallel to advances in our understanding of how extensively the circadian system influences human physiology (12, 13), interest in chronotherapy – the strategic temporal alignment of medical treatment with biological rhythms – has grown rapidly across multiple medical disciplines (14, 15). In bone health, the pharmacodynamics of some osteoporosis medications vary according to the time they are administered, suggesting that certain dosing schedules may optimize therapeutic efficacy or reduce adverse effects (16, 17, 18, 19). With growing interest in how circadian physiology may augment disease development and the effects of existing and emerging endocrine therapies, understanding how intrinsic biological rhythms intersect with bone physiology has increasing clinical relevance.

The objective of this review was therefore twofold:

- To summarize current evidence on the regulation of BTM rhythms, including the relative contributions of endogenous circadian clock mechanisms, behavioral cues, and hormonal signals.
- To synthesize existing clinical and experimental research on chronotherapy in bone health, with a focus on osteoporosis treatments and emerging findings on circadian timing in fracture healing.

## Methods

This narrative review was supported by structured PubMed searches. Two targeted search schemes were performed in PubMed, each aligned with one of the paper’s major sections. One reviewer primarily performed screening and selection, working closely with a second reviewer on most decisions. Results from each search were screened first by title and abstract, followed by full-text review to determine final inclusion. Using backward citation tracking, bibliographies of the included articles were examined to capture any additional studies of relevance. Findings were organized thematically to align with the two major domains of the review.

For the section examining circadian regulation of BTMs, a PubMed search was conducted using the following search string:

(diurnal[tiab] OR circadian[tiab] OR chronobiology[tiab] OR chronotherapy[tiab] OR chronopharmacology[tiab] OR sleep[tiab])

AND (“bone remodeling”[tiab] OR “bone metabolism”[tiab] OR osteoclast[tiab] OR osteoblast[tiab] OR osteocyte[tiab] OR osteoporosis[tiab] OR osteopenia[tiab])

AND (“bone turnover markers”[tiab] OR “bone formation markers”[tiab] OR osteocalcin[tiab] OR P1NP[tiab] OR “bone specific alkaline phosphatase”[tiab] OR BSAP[tiab] OR “bone resorption markers”[tiab] OR CTX[tiab] OR NTX[tiab])

AND human

AND (“2015/01/01”[dp]: “2025/11/01”[dp])

AND (English[lang])

Inclusion criteria comprised relevance to circadian variation in bone physiology and BTMs, human studies, and published between January 1, 2015, and November 1, 2025, to prioritize contemporary evidence (noting that earlier foundational studies were identified and included from the author’s database and through backward citation tracking). Exclusion criteria comprised non-English articles and studies lacking relevance to the domains in inclusion criteria. The search yielded 91 articles, of which 15 were selected for inclusion in this narrative review with structured PubMed searches.

For the section addressing chronotherapy and bone health, two PubMed searches were performed, with the first focused more on clinical conditions and using the following search string:

(chronotherapy[tiab] OR “treatment timing”[tiab] OR “dose timing”[tiab] OR “time of day”[tiab])

AND (osteoporosis[tiab] OR osteopenia[tiab] OR “bone healing”[tiab] OR “bone regeneration”[tiab] OR “fracture repair”[tiab] OR calcitonin[tiab] OR estrogen[tiab] OR “hormone replacement therapy”[tiab])

AND (“2005/01/01”[dp]: “2025/11/01”[dp])

AND (English[lang])

The second chronotherapy search focused on medications and used the following search string:

(diurnal[tiab] OR circadian[tiab] OR chronotherapy[tiab] OR “treatment timing”[tiab] OR “dose timing”[tiab] OR “time of day”[tiab] OR “morning vs evening”[tiab])

AND (teriparatide[tiab] OR abaloparatide[tiab] OR bisphosphonate[tiab] OR alendronate[tiab] OR risedronate[tiab] OR zoledronate[tiab] OR zoledronic[tiab] OR denosumab[tiab] OR romosozumab[tiab] OR raloxifene[tiab] OR “parathyroid hormone”[tiab])

AND (“2005/01/01”[dp]: “2025/11/01”[dp])

AND (English[lang])

Inclusion criteria comprised relevance to clinical implications of chronobiology in bone health, and due to the limited available literature, both chronotherapy searches were broadened to include both human and animal studies in English and encompass publications from January 1, 2005, through November 1, 2025. The chronotherapy topic searches yielded 167 articles, of which 19 were selected for inclusion in this narrative review with structured PubMed searches.

The authors independently prepared the initial manuscript draft. A large language model-based AI tool (ChatGPT, OpenAI) was used at an intermediate drafting stage to suggest alternative wording and improve clarity. All AI-generated suggestions were critically reviewed, edited, or rejected by the authors. The authors verified all scientific content, interpretations, and references and take full responsibility for the final manuscript.

## Circadian regulation of bone turnover marker rhythmicity

### Rhythmicity of bone turnover markers

Diurnal variation refers to the observed pattern of biomarker fluctuation across the 24 h day under real-world conditions, whereas circadian rhythmicity reflects endogenous, near-24 h oscillations generated by intrinsic biological clocks and demonstrated under controlled conditions independent of environmental influences (e.g. constant routine or forced desynchrony protocols). In practice, measured diurnal patterns represent a composite of intrinsic circadian regulation and extrinsic behavioral or environmental influences.

Diurnal variation is a defining feature of biochemical markers of bone turnover and contributes substantially to the variability observed in these measures (1, 3). At the group level, bone resorption markers demonstrate the most pronounced daily oscillations, whereas formation markers generally exhibit smaller amplitude or more variable rhythms (1). Accordingly, this section focuses primarily on the most clinically relevant and widely used BTMs, particularly carboxy-terminal cross-linked telopeptide of type I collagen (CTX) and procollagen type I N-terminal propeptide (P1NP), which are recommended by standard guidelines and commonly employed in both clinical practice and research (20, 21). Understanding these patterns is essential for standardizing biomarker collection to ensure accurate clinical interpretation and for understanding potential implications for chronotherapy and fracture healing.

#### Bone resorption markers

CTX is a degradation fragment released during osteoclastic breakdown of type I collagen in bone (3). CTX can be measured in serum and exhibits one of the strongest diurnal patterns observed among BTMs (1). Several studies have identified a clear nocturnal or early-morning maximum at the group level, followed by a daytime minimum, producing a sinusoidal pattern over the 24 h period (1, 3, 4, 7, 8, 22). The rhythm is consistent across sexes and ethnic groups (23) but varies in amplitude, with older postmenopausal women showing greater peak–trough differences than older men (7).

The pronounced diurnal variation observed in CTX is driven by the internal circadian system and influenced by environmental factors. CTX rhythmicity persists in bed rest studies and blind individuals, but its amplitude is dampened when fasting (1, 3, 8, 24, 25, 26, 27). In clinical practice, variability in CTX levels can be driven by differences in sampling conditions if not controlled. This has led to the recommendation for standardized early-morning fasting sample collection, which minimizes feeding-related variability and improves both intra-individual reproducibility and inter-study comparability. Accordingly, early-morning fasting sampling is considered essential for the accurate clinical use and interpretation of CTX (1, 3, 8, 20, 24).

The N-terminal cross-linked telopeptide of type I collagen (NTX) is a collagen breakdown product, typically assessed in urine, that serves as an indicator of osteoclast-mediated bone resorption (3). Like CTX, it displays a clear diurnal rhythm at the group level, with nighttime peaks and daytime troughs (1, 3). Importantly, NTX rhythms have been shown to persist under constant routine and in individuals without light perception, supporting a contribution of endogenous circadian mechanisms (10, 11). However, these studies also demonstrate important inter-individual variability: not all participants exhibit statistically significant rhythmicity, and among those who do, peak timing can vary widely across the 24 h cycle (10, 11). Accordingly, recommendations for standardized urinary NTX collection – typically using the second morning void – are based less on alignment with a uniform circadian peak and more on practical considerations, including reduction in overnight accumulation effects, minimization of behavioral variability, and improved reproducibility across individuals and studies (28, 29).

Tartrate-resistant acid phosphatase 5b (TRAP5b) is an osteoclast-derived enzyme that reflects osteoclast number rather than collagen degradation (30). The rhythmicity of TRAP5b has not been studied, and its use is currently limited to research only (21).

#### Bone formation markers

Although bone formation markers show daily oscillations at the group level, their amplitudes are generally smaller and more variable than those of resorption markers (3). Therefore, larger sample sizes are often required to detect a statistically significant rhythm, which may explain the variable reporting in the literature (1, 3, 7, 22).

P1NP reflects collagen synthesis and is the preferred formation marker for clinical and research purposes (3, 31). It exhibits a lower-amplitude diurnal variation than CTX or osteocalcin, which may make it easier to use in clinical practice (1, 3, 4). Some studies report no significant 24 h rhythm (7, 22), underscoring its modest amplitude oscillatory pattern and the need for larger sample sizes to reliably detect rhythmicity. Despite this, P1NP consistently shows small nighttime increases in men (7), and across ethnic groups, the rhythm remains unimodal (23).

Osteocalcin (OC), a non-collagenous protein produced by osteoblasts, demonstrates a consistent nighttime or early-morning peak with an afternoon nadir at the group level (1, 3, 4, 7). Because OC is also degraded during bone resorption, its rhythmicity may be more pronounced than that of other bone formation markers, reflecting contributions from both formation and resorption processes (7). This unimodal rhythm has been replicated across ethnically diverse populations (23). OC rhythms are less influenced by food intake than CTX rhythms (3).

Bone-specific alkaline phosphatase (BSAP), another osteoblast-derived marker, demonstrates low-amplitude diurnal fluctuation of less than 15% at the group level (1, 3). This relatively low amplitude, combined with small sample sizes in human studies, likely contributes to variability in BSAP-measured peak times (varying from 12:00 to a wide range of 11:00–20:00 to no rhythmicity) and nadir times (varying from specifically 04:00 to a range of 02:00–06:00 to no rhythmicity) and in overall presence of rhythmic patterns reported between studies (1, 23, 32, 33).

#### Summary

At the overall group level, bone resorption markers exhibit robust, reproducible diurnal rhythms with clear clinical implications for sampling and interpretation, whereas bone formation markers display lower amplitude and more variably detectable diurnal oscillations, underscoring the need to account for marker-specific rhythmicity when designing studies and evaluating time-of-day effects in bone research.

To ensure appropriate interpretation, it is important to note some limitations in the literature. The methodologies used to assess rhythmicity vary considerably across studies, including study design (observational vs constant routine vs forced desynchrony), differences in sampling frequency, and analytical approaches. Many reports derive peak timing from group-averaged data, often from studies with small sample sizes, which may obscure substantial inter-individual variability. Studies demonstrate that, on an individual level, the timing of peak BTM values can vary widely, in some cases spanning much of the 24 h cycle, and that not all individuals exhibit clear rhythmicity (1, 10, 11, 23, 32, 33). As a result, averaging across participants may attenuate or eliminate detectable rhythms, particularly for bone formation markers, potentially leading to underestimation of their rhythmic behavior. In contrast, bone resorption markers may exhibit more consistent timing and larger amplitude rhythms, resulting in more reproducible, consistent findings across populations.

### Clock genes in bone cells

The circadian system is hierarchically organized, with pacemaker neurons in the suprachiasmatic nucleus (SCN) acting as a central clock that coordinates endogenous near-24 h rhythms through conserved molecular clock gene networks, while molecular clocks present in nearly all peripheral tissues regulate local cellular processes and are synchronized by central and environmental signals (12). Bone cells express canonical circadian clock genes, and more than a quarter of bone-expressed genes exhibit circadian rhythms, encompassing transcription factors, cytokines, and signaling proteins that govern osteoblast and osteoclast activity (1). The core clock protein known as brain and muscle aryl hydrocarbon receptor nuclear translocator-like 1 [*ARNTL*] (BMAL1) contributes to normal osteoblast function and engages with metabolic signaling networks, notably those involving insulin (34). In contrast, cryptochrome circadian regulatory protein (CRY2) suppresses osteogenesis; reduced CRY2 permits enhanced circadian locomotor output cycles kaput (CLOCK)- and BMAL1-dependent transcription of osteogenic genes (35). These findings demonstrate that cell-autonomous circadian clocks regulate gene expression within bone cells, thereby influencing osteogenic differentiation and aspects of baseline bone turnover within bone tissue. Conceptually, these peripheral clocks provide tissue-specific timing, but their activity is coordinated by the central circadian pacemaker in the SCN to produce coordinated organism-level rhythms, consistent with hierarchical models of circadian regulation (12).

### Distinguishing endogenous vs exogenous regulation of BTM rhythmicity and clinical implications

A key issue in circadian bone research is determining whether the daily fluctuations in BTMs are driven mainly by the internal circadian clocks or by external influences such as sleep, food intake, posture, light exposure, or hormonal signals. Multiple lines of evidence indicate that endogenous circadian mechanisms are the principal drivers of BTM rhythms and that environmental cues can alter the amplitude. Understanding the relative contributions of intrinsic versus extrinsic factors is essential for developing appropriate interventions.

#### Evidence for intrinsic circadian regulation

The constant routine is an experimental protocol designed to separate endogenous circadian rhythms from environmental cues (e.g. sleep, activity, light, food intake) by eliminating or distributing these cues evenly across a 24 h period (36). It is a preferred study design for isolating intrinsic biological rhythms from environmental influences (11). Under constant routine conditions, urinary NTX demonstrates evidence of endogenous circadian rhythmicity at the group level independent of sleep–wake state, light exposure, posture, or feeding (11). In studies of visually impaired populations, CTX retains its characteristic nocturnal peak (4), and NTX similarly maintains robust 24 h rhythmicity regardless of light perception (10). By contrast, evidence for intrinsic circadian regulation of bone formation markers is more limited; under constant routine conditions, serum CTX exhibits a robust circadian rhythm, whereas the statistical significance of serum P1NP rhythmicity was more variable across participants (37). Similarly, forced desynchrony studies have demonstrated that BTMs, particularly CTX, exhibit robust endogenous circadian rhythmicity that persists independent of sleep–wake timing, feeding schedules, and variations in physical activity (38, 39). These findings support the existence of an intrinsic circadian component to bone turnover regulation.

Melatonin, a hormone secreted by the pineal gland and a marker of the central circadian rhythm, has been implicated in bone metabolism through multiple pathways (40, 41). However, urinary melatonin and urinary NTX often reach their peaks at different times, with evidence showing little to no correlation between their peak timings in both blind and sighted women (10). This indicates that while melatonin may influence bone metabolism, urinary NTX rhythmicity appears to be at least partially driven by intrinsic, peripheral circadian mechanisms rather than the central pacemaker alone.

These findings underscore the robustness of the intrinsic circadian system’s role in bone metabolism, particularly resorption and highlight its potential to modulate the effects of therapeutic interventions.

#### Hormonal modulation

Circulating hormones exhibit their own daily patterns, though current evidence suggests these rhythms modulate rather than generate BTM oscillations. Parathyroid hormone (PTH) exhibits diurnal variation, though with smaller amplitude than CTX or OC (1, 3). PTH regulates serum calcium (3), which is critical for processes such as muscle contraction and cardiac excitability (42). Whether its daily rhythm is purely circadian or influenced by feeding/fasting remains incompletely understood. Furthermore, bimodal PTH rhythms across ethnic cohorts show inconsistent coupling to CTX amplitude (23). Calcitonin also displays daily rhythmicity that parallels BTM fluctuations (1). Some reports on cortisol have shown mixed results linking to OC rhythmicity (4), but other studies demonstrated that cortisol is a major influencer of OC levels (43, 44, 45). In contrast, CTX levels appear unaffected by cortisol variation (43). Endocrine rhythms such as cortisol likely modulate the timing or amplitude of BTM oscillations (46) – especially for bone formation – rather than exerting primary control, which appears to arise predominantly from intrinsic circadian regulatory mechanisms. This distinction highlights how circadian modulators may differentially influence bone formation and resorption.

#### Osteocyte-derived factors

Despite osteocytes’ central role in coordinating bone remodeling, circulating osteocyte-derived factors appear unlikely to serve as primary regulators of systemic BTM rhythmicity, as several factors exhibit little or no circadian rhythmicity (3, 7, 22). Sclerostin, a potent inhibitor of bone formation (47), shows no diurnal variation (7, 22). Other osteocyte markers – including osteoprotegerin (OPG), receptor activator of nuclear factor kappa-B ligand (RANKL), and fibroblast growth factor 23 (FGF23) – either lack diurnal variation or display highly variable timing; (22) however, these markers have not been systematically examined under protocols (e.g. constant routine) that can distinguish intrinsic circadian regulation from environmental or behavioral influences (3, 22).

#### Clinical implications for bone turnover marker measurement

BTM rhythms have direct implications for clinical and research protocols. Standardized sampling conditions – including time of day, fasting status, and specimen type – are essential to minimize pre-analytical variability and ensure comparability across studies and clinical assessments.

As summarized in Table 1, serum CTX requires early morning collection (between 07:30 and 10:00 h) after at least 8 h of overnight fasting (1, 3, 8, 29), whereas urinary NTX should be obtained using a standardized second-morning void protocol (29). P1NP is minimally affected by fasting, shows lower-amplitude circadian variation, allowing more flexibility in sampling and may provide a more consistent measure than CTX if patients are unable to fast prior to collection (3, 20). Although P1NP demonstrates less sensitivity to feeding status and time-of-day compared with CTX, morning sampling remains preferable when markers are interpreted together or followed longitudinally to ensure consistency and comparability (3, 20). OC exhibits marked diurnal variation, and while it is not affected by fasting (3), again, standardized morning sampling minimizes variability related to time-of-day effects.

**Table 1:** Characteristics of bone turnover marker rhythms in humans.

BTM	Bone process	Rhythm amplitude	Peak timing	Preferred collection*
CTX (1, 3, 8, 29)	Resorption	High	Late night/early morning	Morning, fasting
NTX (29)	Resorption	High	Late night/early morning	Urine, second morning void
TRAP5b	Resorption	Unknown	Unknown	--
OC (3)	Formation & resorption	Moderate	Late night/early morning	Morning preferred
P1NP (3, 20)	Formation	Low	Late night/early morning	Flexible, morning preferred
BSAP (1, 3)	Formation	Low/inconsistent	Night/variable	Flexible

* For all bone turnover marker measurements, many laboratories recommend morning fasting for consistency, particularly if concurrently assessed with CTX (21, 29).

BTM, bone turnover marker; CTX, carboxy-terminal cross-linked telopeptide of type I collagen; NTX, N-terminal cross-linked telopeptide of type I collagen; TRAP5b, tartrate-resistant acid phosphatase 5b; OC, osteocalcin; P1NP, procollagen type I N-terminal propeptide; BSAP, bone-specific alkaline phosphatase.

These features inform the National Bone Health Alliance recommendations, which designate fasted-morning CTX and P1NP as standard markers for clinical assessment of bone resorption and formation, respectively (20). Taken together, the circadian properties of BTMs underscore the necessity of time-of-day standardization for reliable interpretation and provide a conceptual framework for understanding how treatment timing may interact with bone physiology, which may be particularly important if BTMs are used as surrogates for fracture risk reduction in clinical trials (48, 49). In practice, this may improve both the reproducibility of research studies and the clinical utility of BTM monitoring.

### Summary and future directions

Taken together, current evidence supports that intrinsic circadian clocks serve as the dominant determinants of bone metabolism, particularly bone resorption marker rhythms. Despite documentation of clock genes in osteoblasts (50), there are less consistent data demonstrating circadian regulation of bone formation markers in humans, possibly due to their lower amplitude rhythms. Behavioral cues, hormonal signals, and osteocyte-derived factors likely function as secondary, modulatory influences. This current conceptual framework provides a foundation for designing both clinical assessments and chronotherapeutic strategies. However, additional research is needed to address outstanding questions, including:

- Understand if and how inter-individual variability in the rhythmicity and peak timing of various BTMs affects bone phenotype, sample collection recommendations, or response to therapy;
- Translate preclinical data on phenotypic bone changes in response to clock gene manipulation into human physiologic relevance to better understand how the central vs peripheral circadian regulation of bone turnover integrates with other known regulators (e.g. sympathetic nervous system, etc.), particularly as it pertains to the coupling, or uncoupling, of bone resorption and formation in humans; and
- Tease apart the relative sensitivities of bone formation and resorption to various environmental cues, and how these sensitivities change over the lifespan, using constant routine and forced desynchrony protocols in humans.

## Chronotherapy and bone health

### Chronotherapy in osteoporosis

Chronotherapy refers to the strategic timing of therapeutic interventions to align with endogenous circadian rhythms in order to optimize efficacy and minimize adverse effects (14). The robust circadian-driven diurnal variation observed in BTMs provides a biological rationale for investigating whether timing of osteoporosis medication administration influences therapeutic efficacy and adverse effect profiles. The following section synthesizes current evidence across anabolic and antiresorptive drug classes to clarify where administration time meaningfully alters treatment efficacy – and where data remain insufficient.

The current evidence base for chronotherapy in prescription osteoporosis medications is heterogeneous and remains limited in scope. Most available studies are small, short-duration investigations that primarily assess surrogate endpoints, particularly changes in BTMs and BMD, rather than fracture. Study designs, populations, and interventions vary substantially, and direct comparisons across therapies are limited. Accordingly, additional, larger trials are needed before routinely implementing chronotherapy data into clinical practice.

#### Anabolic therapies

Parathyroid hormone (PTH) receptor agonists, including teriparatide (PTH 1–34) and abaloparatide (PTH-related peptide analog), are daily injections used to treat osteoporosis by stimulating new bone formation (14). PTH signaling itself exhibits intrinsic temporal specificity: intermittent, pulsatile exposure produces anabolic effects, whereas continuous exposure promotes bone resorption (14). This physiological principle underpins the therapeutic use of these medications, but accumulating evidence shows that the timing of teriparatide administration further modifies its biological impact.

Clinical investigations in postmenopausal women with osteoporosis have shown that administering teriparatide in the morning rather than the evening is associated with more pronounced reductions in circulating CTX and, in some studies, greater gains in lumbar spine BMD; however, these findings are derived from relatively small studies (2, 14, 16, 17, 43). Mechanistic work indicates that exogenous PTH can reset the local circadian clock in cartilage, in a time- and dose-dependent manner (51), raising the possibility that administration time could be investigated as a way to optimize pharmacologic interventions not only in osteoporosis but also in cartilage biology and fracture repair.

Together, these findings suggest that administration time may influence the clinical effects of PTH-based anabolic therapies, with morning dosing producing superior suppression of nocturnal resorption and greater gains in BMD compared with evening dosing. The preferential suppression of nighttime resorption, as reflected by CTX, suggests that the magnitude of teriparatide’s anabolic effect may depend in part on its ability to modify the temporal balance between formation and resorption. For example, teriparatide stimulates bone formation regardless of administration time but the greater suppression of resorption with morning administration results in larger BMD gains because of a larger, more favorable gap between formation and resorption.

Notably, no chronotherapeutic studies have been performed using abaloparatide (i.e. Tymlos). Future research exploring chronotherapeutic effects of abaloparatide and other emerging anabolic therapies (e.g. oral PTH) could clarify whether chronotherapy principles are generalizable across PTH analogs.

#### Antiresorptive therapies

Less robust or clinically applicable evidence exists for antiresorptive osteoporosis therapies, and those with the strongest timing data are limited by safety concerns; nonetheless, their underlying chronotherapeutic effects remain of research interest. Here, we briefly summarize the chronotherapy context for selected antiresorptive agents.

Raloxifene, a selective estrogen receptor modulator (SERM), is FDA-approved for postmenopausal osteoporosis (19). Raloxifene reduces bone alkaline phosphatase and TRAP5b similarly whether taken in the morning or evening (19). However, morning dosing increases plasminogen activator inhibitor (PAI)-1, a biomarker associated with venous thromboembolism risk, whereas evening dosing does not (19). Translation into a clinically meaningful change in venous thromboembolism risk has not been established. Nonetheless, while efficacy appears timing-independent, safety considerations may favor evening administration.

Studies show that calcitonin, uncommonly used for osteoporosis due to limited potency and safety concerns (52), appears to be influenced by administration time – evening dosing suppressed serum CTX by up to 75%, compared with 40–50% when administered in the morning (2, 43, 53). However, one study of oral salmon calcitonin showed that administration timing did not consistently correlate with CTX suppression (54).

Cathepsin K inhibitors (e.g. odanacatib, ONO-5334), are antiresorptive agents shown to improve BMD but are no longer being investigated for osteoporosis due to safety concerns (55, 56). No published chronotherapy data were found for the agent odanacatib, but morning administration of the agent ONO-5334 produced both greater and extended suppression of CTX than evening dosing, potentially enhancing the therapy’s effectiveness in reducing bone loss and fracture risk (18).

Although the long half-lives of other osteoporosis therapies might suggest limited sensitivity to dosing time, even slowly cleared biologics can exhibit circadian variation in pharmacokinetics or target-cell responsiveness (43). However, no studies have examined dosing time effects for monoclonal antibodies such as denosumab and romosozumab, which provide potent antiresorptive and anabolic–antiresorptive effects, respectively (57). Intravenous zoledronic acid, a bisphosphonate used for both osteoporosis and bone-related cancer, shows no significant difference in BTM levels whether administered in the morning or evening; however, these data come from breast cancer patients with 28-day dosing cycles, limiting direct applicability to osteoporosis chronotherapy (58).

#### Summary

**Figure 1:**
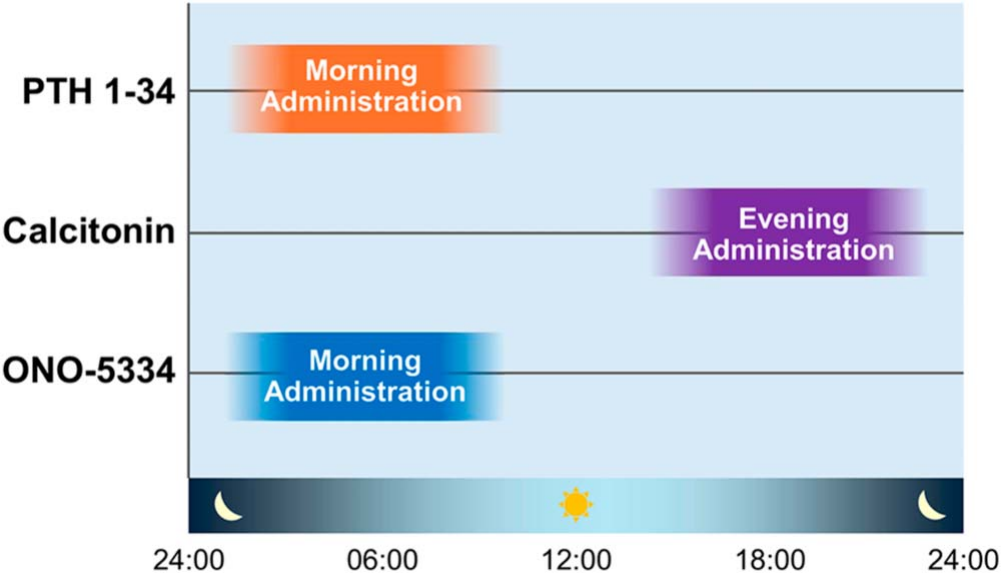
Chronotherapeutic integration across select bone-related medications in humans. Illustrated benefit windows depict the administration time associated with optimal therapeutic effects for selected agents with the most developed or illustrative evidence for time-of-day-dependent effects. Teriparatide (parathyroid hormone 1–34), an anabolic therapy, has the most robust BMD effect when administered in the morning, coinciding with modulation of nocturnal resorption (2, 14, 16, 43). In contrast, the antiresorptive calcitonin (infrequently used due to safety concerns) demonstrates the most robust decrease in CTX when administered in the evening (2, 43, 53). Although not FDA-approved, the cathepsin K inhibitor ONO-5334 (withdrawn from clinical testing, possibly due to class-wide safety concerns after increased incidence of stroke observed in odanacatib clinical trials) (55) showed more sustained suppression of bone resorption markers with morning administration (18). Together, these findings support a circadian-informed framework for optimizing osteoporosis treatment timing, emphasizing that chronotherapeutic effects are drug-specific and based on surrogate (e.g. BTMs, BMD) outcomes, not fracture. Additional therapies, including those with limited, inconsistent, or no demonstrated timing effects, are summarized in Table 2.

Several osteoporosis therapies display chronotherapeutic effects in a drug- and mechanism-specific manner: agents such as teriparatide (PTH 1–34) (2, 14, 16, 17, 43), calcitonin (2, 43, 53), and ONO-5334 (18) show clear time-dependent effects as summarized in Fig. 1, whereas several therapies remain understudied. Figure 1 presents these selected examples as those with the most clearly defined or clinically suggestive timing effects, while a more comprehensive summary of therapies and evidence levels is provided in Table 2. The heterogeneity of these findings underscores the need for drug and class-specific chronotherapeutic frameworks rather than a single universal timing strategy. Overall, while chronotherapy represents a biologically compelling strategy, current evidence does not yet support routine modification of medication timing in clinical practice for most osteoporosis therapies, and further large-scale, outcome-focused studies are needed.

**Table 2:** Evidence for timing effects of bone-related medications in humans.

Agent	Therapy class	Preferred timing	Observed effect	Evidence type/primary endpoint
Teriparatide (PTH 1–34) (2, 14, 16, 43)	Anabolic	Morning	Greater BMD gain, CTX suppression	RCT/biomarker and clinical
Raloxifene (19)	Selective estrogen receptor modulator	Evening	Reduced thrombotic risk markers	RCT/biomarker
Calcitonin* (2, 43, 53)	Antiresorptive	Evening	Greater CTX suppression	RCT/biomarker
ONO-5334^†^ (18)	Antiresorptive cathepsin K inhibitor	Morning	Greater and prolonged CTX suppression	RCT/biomarker
Zoledronic acid (58)	Antiresorptive	None	No clear timing effect	RCT/biomarker and clinical
Abaloparatide, oral bisphosphonates, denosumab, romosozumab			No timing studies	

* Calcitonin is uncommonly used for osteoporosis due to limited potency and safety concerns (52).

^†^ ONO-5334 is not available and no longer being investigated for osteoporosis due to safety concerns (55).

BMD, bone mineral density; CTX, carboxy-terminal cross-linked telopeptide of type I collagen; RCT, randomized controlled trial.

### Clock-modulating therapies

Beyond dosing-time strategies, targeting the molecular clock itself offers another therapeutic avenue. Small-molecule modulators that enhance BMAL1 or influence CRY pathways have been shown in preclinical models to promote osteoblastogenesis or inhibit osteoclastogenesis (2). While still in the experimental stage, these agents act directly on clock-controlled transcriptional pathways in bone cells and are gaining attention as a translational research focus, especially for patients who may have suboptimal responses to standard antiresorptive or anabolic treatments. By fine-tuning the intrinsic circadian machinery of bone, clock modulators could complement traditional pharmacologic interventions and potentially reduce side effects associated with standard treatments.

Melatonin is often used as a physiological marker of the biological night in humans, communicates with peripheral clocks to synchronize them with the central clock, and has been associated with bone metabolism through multiple proposed pathways. Experimental studies show that melatonin can influence bone by promoting osteoblast differentiation (2, 40, 41). Clinical studies in perimenopausal women report increases in femoral neck and spine BMD with melatonin supplementation (2, 59). Melatonin’s dual chronoregulatory and bone-active roles make it a potential adjunct in circadian-aligned osteoporosis therapy.

### Circadian timing in fracture healing

The evidence reviewed above suggests that administration time may modulate the effects of some osteoporosis therapies. These findings raise a broader question: could the same chronobiological principles also apply to the biology of fracture repair? Given that fracture healing recapitulates many components of bone formation, resorption, and remodeling, it is plausible that aligning therapy with endogenous circadian phases may similarly amplify anabolic windows, reduce maladaptive inflammation, and improve clinical outcomes. Supporting this concept, observational data suggest that chronic circadian disruption is associated with impaired skeletal health; for example, long-term rotating night shift work has been linked to increased fracture risk in postmenopausal women (60). These findings provide indirect human evidence that disruption of central circadian regulation may adversely affect bone integrity, reinforcing the potential relevance of circadian timing in fracture repair.

Emerging preclinical data support this conceptual link. Exogenous PTH shifts the peak of circadian activity within murine fracture sites and growth plates (61), suggesting that fracture tissue contains a PTH-responsive local clock. In this model, PTH altered the timing of circadian clock gene oscillations without assessing downstream structural or functional healing outcomes, suggesting a mechanistic effect on clock entrainment rather than proven long-term clinical benefit (61). The timing of PTH delivery may therefore influence not only systemic bone turnover but also the local molecular environment regulating chondrocyte maturation during endochondral ossification, callus mineralization, and remodeling. These observations raise the possibility that peri-operative use of PTH receptor analogs – already of growing interest in orthopedics (61) – could be further optimized by synchronizing dosing with periods of heightened clock-driven responsiveness in injured bone, a hypothesis that warrants direct testing in outcome-focused models.

Beyond osteoporosis-related treatments, other bone-targeted interventions also suggest circadian effects in fracture healing. For example, the timing of non-steroidal anti-inflammatory drug (NSAID) administration can significantly influence fracture healing outcomes (62). NSAIDs are known to impair bone healing under certain conditions, but were found to severely disrupt repair when administered during the resting phase in murine models (62). In contrast, restricting NSAID administration to the active phase was associated with improved healing and recovery, including enhanced molecular activity at the healing callus (62). This suggests that the inflammatory and early reparative stages of fracture healing are circadian-modulated, and that off-phase NSAID administration may disrupt time-sensitive molecular signals important for repair.

Much of the existing evidence on how circadian timing influences fracture healing, although compelling, is derived from rodent models, which exhibit reversed activity cycles (nocturnal behavior) compared with humans, complicating direct alignment of active and resting phases across species. In addition, controlled experimental conditions in animal models do not fully reflect the variability inherent in human trauma, including differences in injury patterns, comorbidities, and treatment timelines.

Practical considerations further limit immediate clinical implementation. In orthopedic and trauma settings, the timing of surgical intervention, medication administration, and postoperative care is often dictated by urgency, resource availability, and patient stability rather than circadian optimization. As such, while these preclinical findings suggest promising avenues for chronobiology-informed strategies, prospective human studies are needed to determine feasibility, optimal timing windows, and clinically meaningful benefits.

While these preclinical findings are promising, the field of circadian fracture therapeutics remains underdeveloped. Virtually, no clinical studies have tested whether the administration time of NSAIDs or PTH receptor analogs influences healing outcomes in humans. Future work should explore whether strategically timed antiresorptives (e.g avoiding nocturnal resorption peaks that might compromise early callus formation) or anabolic agents delivered during the peak bone formation phase can enhance healing rates or reduce complications such as delayed union and nonunion. Additional research could explore whether the timing of surgery – for example, early-morning vs late-afternoon fixation – affects inflammatory cascades, stem cell recruitment, or early mechanotransductive responses at the fracture site. However, it should be noted that surgical timing is usually not a modifiable variable.

Finally, deeper mechanistic studies are needed to map the temporal dynamics of the fracture clock, including whether different stages of healing (inflammation, soft callus, hard callus, remodeling) exhibit distinct circadian-driven rhythms. Defining these time windows could enable development of precision-timed peri-fracture protocols, combining NSAID avoidance strategies, time-targeted PTH receptor analog administration, melatonin, or even tailored nutritional interventions, though these approaches remain theoretical and require further investigation. As osteoporosis chronotherapy continues to advance, extending these principles into fracture repair represents a compelling next frontier for improving musculoskeletal outcomes.

## Conclusion

Bone turnover is governed by intrinsic circadian mechanisms, resulting in predictable, endogenous rhythms in many clinically relevant bone turnover markers – particularly those reflecting bone resorption. Recognition of this temporal structure is essential for accurate biomarker collection, interpretation, and study design and provides the biological foundation for considering time as a modifiable variable in bone health. Behavioral and hormonal factors appear to modulate the amplitude of these rhythms rather than generate them, reinforcing the importance of circadian biology in shaping skeletal metabolism.

Building on this framework, accumulating evidence suggests that aligning osteoporosis therapies with underlying rhythms can meaningfully influence therapeutic response. Chronotherapeutic effects appear most pronounced for agents with short half-lives or pulsatile mechanisms, such as teriparatide, while other treatments remain understudied or relatively insensitive to dosing time, indicating that timing effects are drug-specific. Emerging preclinical data further suggest that circadian principles may extend beyond osteoporosis to fracture healing, although human data are lacking. Future progress will require rigorously designed clinical trials that integrate circadian timing into pharmacologic and surgical paradigms. Incorporating time as a biological variable offers a promising strategy to enhance efficacy, minimize adverse effects, and improve musculoskeletal outcomes without the need for new therapeutic agents.

## Declaration of interest

There is no conflict of interest that could be perceived as prejudicing the impartiality of the research reported.

## Funding

This work was supported by the National Institutes of Healthhttps://doi.org/10.13039/100000002 (R01 HL151332) and VA Eastern Colorado Geriatric Research, Education, and Clinical Center (GRECC).
